# Validity and reliability of the swedish versions of the HLS-EU-Q16 and HLS-EU-Q6 questionnaires

**DOI:** 10.1186/s12889-023-15519-9

**Published:** 2023-04-20

**Authors:** Lina Bergman, Ulrica Nilsson, Karuna Dahlberg, Maria Jaensson, Josefin Wångdahl

**Affiliations:** 1 Department of Neurobiology, Care Sciences and Society, Karolinska Institute, Solna, Sweden; 2 Department of Perioperative Medicine and Intensive Care, Karolinska University Hospital, Stockholm, Sweden; 3 Faculty of Medicine and Health, School of Health Sciences, Örebro University, Örebro, Sweden; 4 Department of Public Health and Caring Sciences, Uppsala University, Uppsala, Sweden; 5 Aging Research Center, Karolinska Institutet & Stockholm University, Tomtebodavägen 18a, Solna, 171 77 Sweden

**Keywords:** Health literacy, HLS-EU-Q, Psychometric study, Validation, Swedish

## Abstract

**Background:**

Health Literacy is a crucial factor for health. In Europe, many people have limited health literacy (i.e. difficulties with accessing, understanding, appraising and using health information). This study aimed to evaluate the psychometrics of the Swedish versions of the HLS-EU-Q16 and HLS-EU-Q6, instruments that aims to assess health literacy.

**Methods:**

In this prospective psychometric study convenience sampling was used, which gave a study population of 347 Swedish-speaking adults. The psychometric evaluation included item distributional statistics, construct validity testing, and principal component analysis to assess structural validity. Internal consistency and test-retest reliability was also investigated.

**Results:**

For the Swedish version of HLS-EU-Q16, no floor effects were detected but a ceiling effect was noted among 28% of the respondents. Construct validity was supported as four out of five expected correlations was confirmed (educational level, self-perceived health, electronic health literacy and HLS-EU-Q6). In terms of structural validity, the principal component analysis yielded a four-factor structure with most items loading significantly only to one factor. The Swedish version of HLS-EU-Q16 had acceptable internal consistency (Cronbach’s α = 0.89, split-half reliability = 0.93) and test-retest reliability showed stability over time (Cohen’s κ = 0.822). For the Swedish version of HLS-EU-Q6, neither floor nor ceiling effects were observed. Construct validity was supported as HLS-EU-Q6 correlated as our a priori stated hypothesis. The principal component analysis did not support the unidimensionality of the scale as a two-factor structure was identified. The Swedish version of HLS-EU-Q6 had acceptable internal consistency (Cronbach’s α = 0.77, split-half reliability = 0.80) and test-retest reliability showed stability over time (Cohen’s κ = 0.812). According to the Swedish version of the HLS-EU-Q16, 71% of the participants were classified as having sufficient comprehensive health knowledge (CHL), while only 33% were classified as having this when the HLS-EU-Q6 was used.

**Conclusions:**

The Swedish versions of the HLS-EU-Q16 and HLS-EU-Q6 have acceptable psychometric properties, and based on the results we recommend its use to measure CHL. However, we are hesitant to use Sw-HLS-EU-Q6 in estimating different CHL levels and further studies need to be conducted to establish validity and accuracy of the thresholds of HLS-EU-Q6.

## Background

Health literacy (HL) is a social determinant of health [1] and one of the three cornerstones of health promotion [2]. In this study we refer to HL as something that “*is linked to literacy and entails people’s knowledge, motivation and competences to access, understand, appraise and apply health information in order to make judgements and take decisions in everyday life concerning health care, disease prevention and health promotion to maintain or improve quality of life during the life course*”(page 3) according to Sorensen et al.’s definition of comprehensive HL (CHL) [3].

In Europe a third to nearly half of the population has low CHL, meaning that at least one in every three people has difficulties with accessing, understanding, appraising, and using health information [4]. The main socio-demographic and socioeconomic factors associated with CHL are age [5], level of education, income, perceived social status [5, 6], and ethnicity [6]. Furthermore, it is evident that there is a social gradient in CHL [1] and that limited CHL can contribute to unequal health [6]. Examples of negative health outcomes that have been associated with limited CHL are difficulties in understanding health information [7], worse preventive behaviours and higher vaccine hesitancy regarding Covid-19 [8], poorer self-perceived general health [5], more visits to general practitioners [5, 9], and longer stays in hospitals [9]. Other examples of associations are less disease knowledge [7, 10], poor self-management behaviours [10], and poor medical adherence [7].

Tailored interventions to improve HL can result in increased empowerment and improved decision-making skills, thus helping people take a more active part in their treatment and care [11, 12]. However, in order to measure if the intervention has a positive effect on HL, reliable and valid HL questionnaires are needed [13]. The Health Literacy Survey European Questionnaire, HLS-EU-Q, is a self-reporting instrument consisting of 47 items that was developed by Sørensen et al. in 2012. It is based on a systematic literature review that obtained an all-inclusive conceptual model along with Sorensen’s definition of CHL [3] as used in this study. The original English version of the HLS-EU-Q47 has been translated into more than 20 languages [14]. Two shorter and less time-consuming versions, HLS-EU-Q16 (3 min) and HLS-EU-Q6 (1 min), have been constructed from the HLS-EU-Q47. The HLS-EU-Q16 consists of 16 of the 47 items in the HLS-EU-Q47 that were selected using a one-parametric dichotomous Rasch model. The HLS-EU-Q6 consists of 6 items from the HLS-EU-Q16 that were selected based on results from a confirmatory factor analysis modelling and on higher item difficulty [15].

Several language versions of the HLS-EU-Q16 have been evaluated for its psychometric properties, showing that it is psychometrically sound [16–21], although the Japanese version of HLS-EU-Q16 had weak validity [20]. Psychometric evaluations of the short-short form, the HLS-EU-Q6, are few and with diverse results [18, 21]. The Italian version has been found to be reliable and valid [18], but the validity of the French version could not be established [21]. The Swedish version of the HLS-EU-Q16 (Sw-HLS-EU-Q16) was adapted and translated in line with guidelines for the translation of questionnaires [22] and so far used in one study including a Swedish speaking population [23]. However, it has not yet been psychometrically evaluated. The Swedish HLS-EU-Q6 (Sw-HLS-EU-Q6) has not been used in any study nor has it been validated. It is therefore important to examine the validity and reliability of the of Sw-HLS-EU-Q16 and Sw-HLS-EU-Q6.

## Methods

### Aim

The aim of this study was to psychometrically evaluate the Swedish versions of the HLS-EU-Q16 and HLS-EU-Q6 as well as to investigate the response patterns.

### Study design

This prospective psychometric evaluation study is a part of a research project aiming to measure electronic health literacy and CHL in a population of Swedish and Arabic-speaking persons in Sweden [24–28].

### Setting, sample, and data collection

This study aimed for a target sample of 300 participants as informed by the recommended sample size for psychometric validation studies [29]. The inclusion criteria were ≥ 18 years of age, having Swedish as a native language, and being available on the day of the data collection. The participants were recruited by convenience sampling from university courses, craft training, larger workplaces with academic and non-academic staff, non-governmental organisations serving elderly people, athletic clubs, and two choirs. The arenas included a diversity of groups of different ages, sexes, and levels of education. The different arenas selected for recruitment were visited at one or more time points during the data collection period. Potential participants were informed verbally and in writing about the study design by either the last author (JW) or by key stakeholders (i.e., organisation managers or others) selected by the researchers.

In the test-retest analysis 35 participants were invited to answer the questionnaire twice within one week. A sample size of at least 25 participants in the retest was deemed applicable [30]. To compare answers from the test and retest on an individual level, the participants marked their questionnaires with a code comprising the first three letters of their mother’s name and the year she was born. The data collection was carried out from February to May 2019.

### Questionnaires

The Sw-HLS-EU-Q16 is a self-reported tool with Likert-type responses (“very easy”, “easy”, “difficult”, “very difficult”) and an associated sum score that measures individuals’ ability to access, understand, appraise, and apply health information. An overall Sw-HLS-EU-Q16 index score was calculated in three steps according to the developer [15, 31]. First, the response categories for the 16 items were dichotomized into easy (very easy and easy) with a value of 1 and difficult (difficult and very difficult) with a value of 0. Second, an overall index score was calculated by adding all the values obtained. Third, the index score was divided into three categories: inadequate (0–8 score points), problematic (9–12 score points), and sufficient (13–16 score points) CHL. To calculate the HLS index score, the respondent needs to have answered at least 14 of the 16 items.

To produce the score of the Sw-HLS-EU-Q6, the response categories were not dichotomised as for the Sw-HLS-EU-Q16 according Pelikan et al. 2014 [15]. Instead, the value for the response category was used: “very easy” = 4; “easy” = 3; “difficult” = 2; “very difficult” = 1; “don’t know/refusal” = missing [15]. First, the index score was calculated by adding all the values obtained and dividing by the number of items (requires response on at least 5 of the 6 items). Second, the index score was divided into three categories: inadequate CHL (≤ 2 score points), problematic CHL (> 2 and ≤ 3 score points), and sufficient CHL (score > 3) CHL. The cut-off values used for determining the three CHL levels for the Sw-HLS-EU-Q16 and Sw-HLS-EU-Q6 were the same as those recommended by Pelikan et al. [15] and that were used in Rouquette et al.’s study [21]. In addition, socio-demographic data (age, biological sex, and education) were collected.

### Psychometric testing and data analysis

The inclusion criterion was a valid index score for the HLS-EU-Q16. Data are presented as mean, SD, number, percentage (%), or range. All percentages are rounded up to the nearest integer. The psychometric testing was guided by the COnsensus-based Standards for the selection of health Measurement INstruments (COSMIN) and its recommendations for analysis when using classical test theory [32].

**Floor and ceiling effects** were examined on item and CHL score level by calculating the percentage of lowest or highest possible score received by the respondents and were considered acceptable if < 15% scored at the floor or ceiling [33]. Frequency of missing data was evaluated toward the criteria of < 5% [34].

**Construct validity** considered how the instruments correlated with other instruments or variables [35].Spearman’s rank coefficient was used to analyse the correlation between Sw-HLS-EU-Q16 index score, Sw-HLS-EU-Q6 index score, age, level of education, self-perceived health, and Swedish Electronic Health Literacy Scale (Sw-eHEALS) [24] sum score. Sw-eHEALS measures electronic HL, i.e. HL skills in relation to online information and applications [36, 37]. General self-perceived health was assessed with the question *How do you assess your overall health status*, and the response categories were very poor, poor, fair, good, or very good [27]. Based on our previous research [24, 25], we hypothesised that the Sw-HLS-EU-Q16 and Sw-HLS-EU-Q6 should be negatively correlated with high age and positively correlated with high level of education, high self-perceived health, and high score on Sw-eHEALS as well as positively correlated with each other (i.e. higher HL score on the Sw-HLS-EU-Q16 would imply higher HL score on the Sw-HLS-EU-Q6). A correlation coefficient magnitude between 0 and 0.1 was negligible, between 0.1 and 0.39 was weak, between 0.4 and 0.69 was moderate, between 0.7 and 0.89 was strong, and between 0.9 and 1.0 was very strong [38]. Structural validity refers to the extent to which the structure of the instrument adequately reflects the hypothesised dimensionality of the construct being measured [34]. In line with previous research [16, 39] evaluating the validity of HLS-EU-Q16, principal component analysis (PCA) was performed. The Kaiser-Meyer-Olkin index (criteria > 0.8) and Bartlett’s test of sphericity (p < 0.05) was used to assess appropriateness of the data set. Factor extraction was based on eigenvalues > 1 and visual examination of the scree-plot. The factor solution was rotated using oblique (oblimin) rotation [40].

**Test-retest reliability**, analyses the instruments consistency over time [33] and was analysed with weighted quadratic Cohen’s κ coefficient in order to measure the agreement between the two time points. A value of ≥ 0.70 was considered acceptable [41].

**Internal consistency reliability** is *“the interrelatedness among the items”* (page 2. Mokkink et al., 2010) [35]. It was assessed using Cronbach’s α and split-half reliability was calculated using Spearman–Brown’s coefficient with a reliability coefficient of 0.70–0.95 considered acceptable. To investigate **response patterns** between participants with valid Sw-HLS-EU-Q16 HL scores and those without, differences in sex were analysed with Chi-square test and differences in age were analysed with Student’s t-test. Differences in age, educational levels, general self-perceived health, Sw-HLS-EU-Q16, and Sw-HLS-EU-Q6 levels between participants were analysed with the Wilcoxon signed***-***rank test. The Mann–Whitney U-test was used to analyse differences in educational level. Two-tailed *P*-values under 0.05 were considered to be statistically significant.

## Results

### Demographics of the sample

A total of 347 respondents participated. Of these, 12 (3.5%) were excluded due to no valid CHL score for the Sw-HLS-EU-Q16 (i.e. >2 missing items). There was a significant difference in age between included and excluded participants who did not have a valid Sw-HLS-EU-Q16 index score (mean age 48.7 vs. 69.4, p = 0.001), but there were no significant differences in regard to gender (p = 0.274) or educational level (p = 0.895). No structural patterns in terms of difficulty in responding to certain items were observed.

For the total included sample, the mean age was 48.7 years (range 19–98 years) and around half were females (n = 168; 51%). The majority had at least 10 years of education (n = 303; 91%) and perceived their own general health to be good or very good (n = 290; 87%). Accordingly, for the Sw-HLS-EU-Q16 a total of 239 (71%) participants had sufficient CHL levels, and for the Sw-HLS-EU-Q6 a total of 112 (33%) participants were classified as having sufficient CHL (Table 1).

**Table 1:** Demographics of the test group (n = 335) and of the test-retest group (n = 38)

Characteristics	Total sample^a^	Test-retest group
**Biological sex, n (%)**		
Man	164 (49)	15 (40)
Woman	168 (51)	23 (60)
**Age in years**		
Mean (SD)	48.7 (21.4)	46.0 (26.0)
Range	19–94	26–89
**Highest education level, n (%)**		
1–6 years	3 (1)	0 (0)
7–9 years	26 (8)	5 (13)
10–12 years	156 (47)	4 (11)
Graduated from university	147 (44)	29 (76)
**General self-perceived health, n (%)**		
Very poor	0 (0)	0 (0)
Poor	7 (2)	1 (3)
Fair	38 (11)	2 (5)
Good	203 (61)	26 (68)
Very good	87 (26)	9 (24)
**Sw-HLS-EU-Q16**^**b**^, **n (%)**		
Inadequate	21 (6)	3 (8)
Problematic	75 (22)	4 (11)
Sufficient	239 (71)	31 (82)
Mean index score (SD)	13.5 (2.6)	13.9 (2.8)
Range	5–16	6–16
**Sw-HLS-EU-Q6**^**c**^, **n (%)**		
Inadequate	11 (3)	1 (3)
Problematic	212 (63)	20 (53)
Sufficient	112 (33)	17 (45)
Mean index score (SD)	2.9 (0.5)	3.0 (0.5)
Range	1.5–4	2–4
**Sw-eHEALS** ^**d**^		
Mean sum score (SD)	29.5 (5.9)	32.6 (6.1)
Range	8–40	12–40

^a^Missing responses (biological sex, n = 3; educational level, n = 3; Sw-eHEALS, n = 21) were not included in the denominator when calculating percentages; ^b^The Swedish Health Literacy Survey European Questionnaire 16 items; ^c^The Swedish Health Literacy Survey European Questionnaire 6 items; ^d^The Swedish eHealth Literacy Scale

The retest group consisted of 38 respondents. The mean age was 46 years (range 26–89 years), 23 (60%) were females, the majority had graduated from university (n = 29; 76%), and 35 (92%) perceived their general health as good or very good. The respondents’ mean index score for Sw-HLS-EU-Q16 was 13.9 (SD 2.8), and 31 (82%) were classified as having sufficient CHL. For the Sw-HLS-EU-Q6, the respondents had a mean index score of 3 (SD 0.5), and 17 (45%) were classified as having sufficient CHL (Table 1).

### Item distribution statistics and floor and ceiling effects

Table 2 shows the distribution statistics for each item on the Sw-HLS-EU-Q16 and Sw-HLS-EU-Q6. No floor effects were observed on the item level, and the proportion of respondents scoring the lowest possible score was < 15%. All items except “*judge when you may need to get a second opinion from another doctor*” and “*decide how you can protect yourself from illness based on information in the media*” showed ceiling effects, and the proportion of respondents scoring the highest possible score was > 15%. Most of the items (13/16) had full variance (i.e. at least one respondent for each scoring option), and missing values were < 1% (n = 3) (Table 2).

**Table 2:** Distributional statistics for individual Sw-HLS-EU-Q16/Q6 items

Items, n (%)	Very difficult	Difficult	Easy	Very easy	Missing
A. Find information on treatments of illnesses that concern you	2 (1)	33 (10)	216 (64)	82 (24)	2 (1)
B. Find out where to get professional help when you are ill	1 (0)	44 (13)	184 (55)	105 (31)	1 (0)
C. Understand what your doctor says to you	0 (0)	24 (7)	209 (62)	100 (30)	2 (1)
D. Understand your doctor’s or pharmacist’s instruction on how to take a prescribed medicine	0 (0)	9 (3)	153 (46)	173 (52)	0 (0)
**E. Judge when you may need to get a second opinion from another doctor**	**21 (6)**	**129 (39)**	**139 (41)**	**44 (13)**	**2 (1)**
**F. Use information the doctor gives you to make decisions about your illness**	**3 (1)**	**67 (20)**	**196 (59)**	**67 (20)**	**2 (1)**
G. Follow instructions from your doctor or pharmacist	0 (0)	7 (2)	170 (51)	157 (47)	1 (0)
**H. Find information on how to manage mental health problems like stress or depression**	**14 (4)**	**95 (28)**	**172 (51)**	**52 (16)**	**2 (1)**
I. Understand health warnings about behaviour such as smoking, low physical activity and drinking too much	1 (0)	13 (4)	131 (39)	189 (56)	1 (0)
J. Understand why you need health screenings	2 (1)	17 (5)	138 (41)	176 (53)	2 (1)
**K. Judge if the information on health risks in the media is reliable**	**8 (2)**	**102 (30)**	**165 (49)**	**60 (18)**	**0 (0)**
L. Decide how you can protect yourself from illness based on information in the media	5 (1)	89 (27)	193 (58)	47 (14)	1 (0)
**M. Find out about activities that are good for your mental well-being**	**1 (0)**	**32 (10)**	**185 (55)**	**116 (35)**	**1 (0)**
N. Understand advice on health from family members or friends	2 (1)	34 (10)	190 (57)	107 (32)	2 (1)
**O. Understand information in the media on how to get healthier**	**4 (1)**	**49 (15)**	**190 (57)**	**89 (27)**	**3 (1)**
P. Judge which everyday behaviour is related to your health	3 (1)	18 (5)	173 (52)	138 (41)	3 (1)

Bold text: items included in Sw-HLS-EU-Q6

Sw-HLS-EU-Q16: A ceiling effect was noted with a total of 94 (28%) respondents scoring the maximum CHL index score of 16. No floor effects were detected, and no respondents received the minimum CHL index score of 0. Sw-HLS-EU-Q6: No floor or ceiling effects were noted, and a total of 11 (3%) respondents scored the maximum CHL index score of 4 and no respondents received the minimum CHL index score of 1.

### Construct and structural validity

Sw-HLS-EU-Q16 was statistically weakly positively correlated with higher education level and higher self-perceived health, moderately positively correlated with higher Sw-eHEALS sum score, and strongly positively correlated with higher Sw-HLS-EU-Q6 index score. The Sw-HLS-EU-Q6 was also weakly positively correlated with higher education level and higher self-perceived health, moderately positively correlated with higher Sw-eHEALS sum score, and strongly positively correlated with higher Sw-HLS-EU-Q16 index score. No correlations were found between age and the Sw-HLS-EU-Q16 or Sw-HLS-EU-Q6. The Spearman correlation between the Sw-HLS-EU-Q16 and Sw-HLS-EU-Q6 scores was 0.84 (Table 3).

**Table 3:** Spearman rho correlations between Sw-HLS-EU-Q16, Sw-HLS-EU-Q6, age, education, self-perceived health and Sw-eHEALS

Variables	1.	2.	3.	4.	5.	6.
1. Sw-HLS-EU-Q16^a^	1					
2. Sw-HLS-EU-Q6^b^	0.840**	1				
3. Age^c^	0.071	0.040	1			
4. Educational level^d^	0.135*	0.157**	0.095	1		
5.Self-percieved health^e^	0.167**	0.177**	− 0.153**	0.145**	1	
6. Sw-eHEALS sum score^f^	0.496**	0.551**	− 0.273**	0.238**	0.177**	1

^a^Sw-HLS-EU-Q16 index score, range 5–16; ^b^Sw-HLS-EU-Q6 index score, range 1.5–4; ^c^Age, range 19–94; ^d^Highest educational level, 1 = none, 2 = 1–6 years, 3 = 7–9 years, 4 = 10–12 years, 5 = Graduated from university; ^e^Self-perceived health, 1 = very poor, 2 = poor, 3 = fair, 4 = good, 5 = very good; ^f^Sw-eHEALS sum score, range 8–40

* Correlation is significant at the 0.05 level (2-tailed) ** Correlation is significant at the 0.01 level (2–tailed). Abbreviations: Sw-HLS-EU-Q: Swedish Health Literacy Survey European Questionnaire; Sw-eHEALS: Swedish eHealth Literacy Scale

For Sw-HLS-EU-Q16, the PCA yielded a four-factor model based on eigenvalues > 1 (KMO index value 0.890; Barlett’s test of sphericity < 0.001; variance explained 60.6%). However, in interpreting the scree-plot, a one factor solution could also be considered as a final structure. For the four-factor model, the items that clustered together suggest that factor 1 (item o, l, n, k, m and p) represents “Access, understand and process information in relation to health”; factor 2 (item d, g, c and j) represents “Understand information and follow instructions from healthcare professionals”; factor 3 (item e and f) represents “Process and apply information from healthcare”; and factor 4 (item a, b, I and h) represents “Access and understand information in relation to illness). All items loaded significantly (> 0.4) to only one factor except item J (*Understand why you need health screenings)* that had no significant loading and item K (*Judge if the information on health risks in the media is reliable)* that cross loaded on factor 1 and 3 (Table 4).

**Table 4:** Factor loadings of Swe-HLS-EU-Q16 (PCA, Oblimin rotation, n = 335)

Item	Factor				Subscale
	1	2	3	4	
O. Understand information in the media on how to get healthier	**0.844**	0.031	− 0.016	− 0.033	Access, understand and process information in relation to health
L. Decide how you can protect yourself from illness based on information in the media	**0.756**	− 0.124	− 0.329	0.023	
N. Understand advice on health from family members or friends	**0.671**	0.109	0.208	0.030	
K. Judge if the information on health risks in the media is reliable	**0.598**	− 0.010	− 0.417	0.017	
M. Find out about activities that are good for your mental well-being	**0.466**	0.046	0.227	0.386	
P. Judge which everyday behaviour is related to your health	**0.448**	0.263	0.108	0.116	
D. Understand your doctor’s or pharmacist’s instruction on how to take a prescribed medicine	− 0.038	**0.913**	− 0.040	− 0.055	Understand information and follow instructions from healthcare professionals
G. Follow instructions from your doctor or pharmacist	0.016	**0.868**	− 0.001	− 0.042	
C. Understand what your doctor says to you	0.057	**0.610**	− 0.305	0.080	
J. Understand why you need health screenings	0.129	**0.351**	0.170	0.275	
E. Judge when you may need to get a second opinion from another doctor	0.092	0.140	**− 0.690**	0.146	Process and apply information from healthcare
F. Use information the doctor gives you to make decisions about your illness	0.041	0.315	**− 0.574**	0.234	
A. Find information on treatments of illnesses that concern you	− 0.112	− 0.050	− 0.124	**0.851**	Access and understand information in relation to illness
B. Find out where to get professional help when you are ill	0.044	0.049	− 0.081	**0.700**	
I. Understand health warnings about behaviour such as smoking, low physical activity and drinking too much	0.052	0.088	0.269	**0.665**	
H. Find information on how to manage mental health problems like stress or depression	0.111	− 0.045	− 0.194	**0.660**	

Abbreviations: Sw-HLS-EU-Q: Swedish Health literacy survey European questionnaire; PCA: Principal Component Analysis

For Sw-HLS-EU-Q6, the PCA yielded a two-factor model based on eigenvalues > 1 (KMO index value 0.781; Barlett’s test of sphericity < 0.001; variance explained 64.5%). The items that clustered together suggest that factor 1 (item m, o and h) represents “Access and understand information in relation to health and illness” and factor 2 (item e, f and k) represent “Process and apply information from healthcare professionals and media”. All items loaded significantly only to one factor (Table 5).

**Table 5:** Factor loadings of Swe-HLS-EU-Q6 (PCA, Oblimin rotation, n = 335)

Item	Factor		Subscale
	1	2	
M. Find out about activities that are good for your mental well-being	**0.885**	0.146	Access and understand information in relation to health and illness
O. Understand information in the media on how to get healthier	**0.800**	− 0.035	
H. Find information on how to manage mental health problems like stress or depression	**0.597**	− 0.234	
E. Judge when you may need to get a second opinion from another doctor	− 0.124	**− 0.927**	Process and apply information from healthcare professionals and media
F. Use information the doctor gives you to make decisions about your illness	0.071	**− 0.786**	
K. Judge if the information on health risks in the media is reliable	0.366	**− 0.505**	

Abbreviations: Sw-HLS-EU-Q: Swedish Health literacy survey European questionnaire; PCA: Principal Component Analysis

### Internal consistency

Cronbach’s α for the Sw-HLS-EU-Q16 and Sw-HLS-EU-Q6 was 0.89 and 0.77, respectively, and the split-half reliability computed by Spearman–Brown’s coefficient for the Sw-HLS-EU-Q16 and Sw-HLS-EU-Q6 was 0.93 and 0.80, respectively (Table 6).

**Table 6:** Test-retest reliability statistics for the Sw-HLS-EU-Q16 and Sw-HLS-EU-Q6, (n = 38)

Variables	Cronbach’s α	Spearman-Brown’s coefficient	Cohen’s κ ^a^
Sw-HLS-EU-Q16	0.890	0.929	0.822*
Sw-HLS-EU-Q16 levels			0.875*
Sw-HLS-EU-Q6	0.777	0.801	0.812*
Sw-HLS-EU-Q6 levels			0.626*

* Significant at the 0.01 level (2–tailed)

### Test-retest reliability

Test-retest reliability of the Sw-HLS-EU-Q16 index score, Sw-HLS-EU-Q6 index score, and Sw-HLS-EU-Q16 levels was acceptable with Cohens κ > 0.7. For Sw-HLS-EU-Q6 levels, Cohen’s κ was 0.63 (Table 6). On the item level, the test-retest reliability was acceptable for 2 of 16 items (Cohen’s κ = 0.21–0.74).

## Discussion

Our results indicate that the Swedish version of the HLS-EU-Q16 is a valid instrument, but the validity of the Swedish version of HLS-EU-Q6 is questionable. A higher proportion of participants were classified as having sufficient CHL when the HLS-EU-Q16 was used than when the HLS-EU-Q6 was used, namely 71% vs. 33%, respectively, in our Swedish version. This has also been described in the French version where 58% (HLS-EU-Q16) vs. 26% (HLS-EU-Q6) had sufficient CHL [21]. The discrepancy between the two versions in an Italian validation study was smaller at 33.0% vs. 24.6% [18], respectively. However, the proportion of participants with sufficient CHL assessed with the HLS-EU-Q6 was still much smaller compared with both our Swedish study and the French validation study [21]. The low proportion of participants with sufficient CHL when using the HLS-EU-Q16 was noticed by the authors themselves who suggested that the differences could be due to aspects such as cultural norms, the role of the family, and the usability of media sources in the specific area in the country [18]. Furthermore, a low proportion of participants with sufficient CHL was identified in the validation of the Brazilian Portuguese version of HLS-EU-Q6, in which only 2% of the participants were classified as having sufficient levels of CHL [42]. By examining how the participants in our study responded at the item level on the Sw-HLS-EU-Q16, we found that five of the items (items E, F, H, K, O) that had been most frequently responded to as being difficult or very difficult are included in Sw-HLS-EU-Q6. This is probably the main reason for why a higher proportion of participants were classified as having lower CHL when using the Sw-HLS-EU-Q6 than when using the Sw-HLS-EU-Q16. However, there was a strong correlation between the Sw-HLS-EU-Q6 and Sw-HLS-EU-Q16 (r = 0.84) even though the majority of the six items included in the Sw-HLS-EU-Q6 were scored lower than other items in the Sw-HLS-EU-Q16. The reason for this strong correlation can be explained by the fact that a correlation analysis does not take into account the interval between the variables [33]. [33] The constructer of the HLS-EU-Q6 also found strong correlations with the HLS-EU-Q47 (r = 0.90) and HLS-EU-Q16 (r = 0.82) [43]. The constructors (Jurgen Pelikan, personal communication 22/11/2021) further stated that reduction from 16 to 6 items led to significant loss of information and loss of representativeness of the theoretical scope [15] and thus it was not recommended to construct levels for the HLS-EU-Q6. The authors of the French version came to the same conclusion [21].

The discrepancy in CHL levels between the two HLS-EU versions is problematic, especially when comparing and implementing results and when drawing conclusions from the specific population. Moreover, the results from the analyses regarding internal consistency, validity, and test-retest reliability of the Sw-HLS-EU-Q6 were not conclusive. On one hand, no ceiling effects were noted, and construct validity as well as internal consistency were acceptable. However, our results showed that the Sw-HLS-EU-Q6 was not unidimensional as the PCA yielded a two-factor model. Thus, in this stage we, in line with the authors of the French validation study [21], do not recommend using the HLS-EU-Q6. In order to be able get a short version of the HLS-EU-Q47 such as HLS-EU-Q6, further work is needed in selecting appropriate items and revision of the thresholds described by Pelikan et al. [15]. Furthermore, comparisons of the distribution of CHL levels between the Sw-HLS-EU-Q16 and Sw-HLS-EU-Q6 with a Swedish version of the HLS-EU-Q47 would be valuable. However, there is yet no Swedish version of the latter.

Our results indicate that the psychometric properties of the Sw-HLS-EU-Q16 are acceptable in terms of internal consistency, test-retest reliability and construct validity. Our results are in line with previous studies, for example, the Sw-HLS-EU-Q16 had a Cronbach’s α = 0.89, which is in line with results from the French [21, 44], Icelandic [16], and Indian [19] versions of the HLS-EU-Q16. On the other hand, a ceiling effect of 28% was observed. This ceiling effect was also observed in the two French validation studies of the HLS-EU-Q16, with values of 21% [43] and 25% [21]. In our study all items except “*judge when you may need to get a second opinion from another doctor*” and “*decide how you can protect yourself from illness based on information in the media*” showed ceiling effects. This skewed distribution may indicate a country-specific limitation, which may affect the accuracy of the questionnaire. In terms of structural validity, the factor structure identified in the present study is in line with the Islandic [16] and Romanian versions [39], but factor loading pattern of the Sw-HLS-EU-Q16 differed from the original model as well as with the two previous mentioned studies. The results from the present study and previous research indicate that neither the domains nor the competencies of CHL that underlie the questionnaire manifest the same way across cultures [5]. We therefore only recommend calculating a sum score when using the Sw-HLS-EU-Q16.

Interestingly, 90% of the participants in our study thought it was easy/very easy.

“*to find information on treatments of illnesses”*. It is known that patients or relatives perform searches on the internet for information before and after clinical consultation in order to obtain more information and to make their own decision on the suggested diagnosis and treatment [45]. However, whether the person can assess if the information obtained is correct and reliable is debatable. On the other hand, the findings from our study indicate that the participants seem to have trust in and understand the instructions from health care, and a high proportion of the participants thought it was easy/very easy to: “*understand your doctor’s or pharmacist’s instruction on how to take a prescribed medicine*” (98%), “*follow instructions from your doctor or pharmacist*” (98%), “*understand why you need health screenings*” (94%), and *“understand what your doctor says to you*” (93%). These results are in line with the results from the validation of the Italian version of the HLS-EU-Q16 [18].

This study has some limitations to note. We aimed for a study population that included groups of different ages, genders and educational levels. However, it ended up that most of the participants had at least 10 years of education (many of whom also had some form of university education). This makes it difficult to assess whether the results of the study also apply to low-educated people in general. However, the overall results from the cognitive interviews conducted in a previous study to test the content validity of the instrument indicate that the instrument could be understood by less educated people even if it was not completely easy (unpublished results). A study from the Netherlands that shows that the instrument can be valid even among low-educated people show similar results. This as it also highlights that it would be good if some wordings were simplified, and extra contextual information were added [46]. Furthermore, we used a sample of participants from the capital of Sweden and a nearby city, areas that may not be representative of the majority of Swedish speakers. Our results must therefore be replicated on samples of subjects from other parts of Sweden before anything can be said about their robustness, especially in rural Sweden. Moreover, the use of self-administered questionnaires is restricted to people who can read and understand Swedish, and the HLS-EU-Q16 measures self-reported CHL, which is not the same as measuring the person’s knowledge of health.

Furthermore, answering self-reported HL questionnaires has been criticized for the cognitive burden imposed on the respondents [16, 47]. It would also be of value to validate the instruments in a study where data were collected using face-to-face interviews because this is how the instrument originally was used. This would include people with limited functional HL who might have difficulties with filling in forms [48]. Still, there were very few missing answers, and only 3.5% of the questionnaires were excluded due to no valid CHL score for the Sw-HLS-EU-16. Based on these limitations, we suggest that other methods should be combined with these subjective methods, such as interviews and observations, in order to increase the authenticity and objectivity of the data.

## Conclusion

The Swedish version of the HLS-EU-Q6 and HLS-EU-Q16 have acceptable psychometric properties, and based on these results they can be used to measure CHL. However, we are hesitant to use Sw-HLS-EU-Q6 in estimating different CHL levels, as the agreement with the Sw-HLS-EU-Q16 when dividing the CHL index into inadequate, problematic and sufficient was poor. Further studies need to be conducted to establish validity and accuracy of the thresholds of HLS-EU-Q6. In addition, we recommend further studies comparing the Swedish short versions with the HLS-EU-Q47, i.e., the original version of the instrument and other instruments measuring CHL.

## Data Availability

The datasets used and/or analysed during the current study are available from the corresponding author on reasonable request.

## List of abbreviations

CHL: Comprehensive health literacy
COSMIN: The COnsensus-based Standards for the selection of health Measurement Instruments
HLS-EU-Q: European Health Literacy Survey Questionnaire
KMO: Kaiser-Meyer-Olkin
PCA: Principal Component analysis
Sw-HLS-EU-Q: Swedish European Health Literacy Survey Questionnaire
Sw-eHEALS: Swedish Electronic Health Literacy Scale
